# *VKORC1* Common Variation and Bone Mineral Density in the Third National Health and Nutrition Examination Survey

**DOI:** 10.1371/journal.pone.0015088

**Published:** 2010-12-13

**Authors:** Dana C. Crawford, Kristin Brown-Gentry, Mark J. Rieder

**Affiliations:** 1 Center for Human Genetics Research, Vanderbilt University, Nashville, Tennessee, United States of America; 2 Department of Molecular Physiology and Biophysics, Vanderbilt University, Nashville, Tennessee, United States of America; 3 Department of Genome Sciences, University of Washington, Seattle, Washington, United States of America; Harvard University, United States of America

## Abstract

Osteoporosis, defined by low bone mineral density (BMD), is common among postmenopausal women. The distribution of BMD varies across populations and is shaped by both environmental and genetic factors. Because the candidate gene vitamin K epoxide reductase complex subunit 1 (*VKORC1*) generates vitamin K quinone, a cofactor for the gamma-carboxylation of bone-related proteins such as osteocalcin, we hypothesized that *VKORC1* genetic variants may be associated with BMD and osteoporosis in the general population. To test this hypothesis, we genotyped six *VKORC1* SNPs in 7,159 individuals from the Third National Health and Nutrition Examination Survey (NHANES III). NHANES III is a nationally representative sample linked to health and lifestyle variables including BMD, which was measured using dual energy x-ray absorptiometry (DEXA) on four regions of the proximal femur. In adjusted models stratified by race/ethnicity and sex, SNPs rs9923231 and rs9934438 were associated with increased BMD (p = 0.039 and 0.024, respectively) while rs8050894 was associated with decreased BMD (p = 0.016) among non-Hispanic black males (n = 619). *VKORC1* rs2884737 was associated with decreased BMD among Mexican-American males (n = 795; p = 0.004). We then tested for associations between *VKORC1* SNPs and osteoporosis, but the results did not mirror the associations observed between *VKORC1* and BMD, possibly due to small numbers of cases. This is the first report of *VKORC1* common genetic variation associated with BMD, and one of the few reports available that investigate the genetics of BMD and osteoporosis in diverse populations.

## Introduction

The candidate gene vitamin K epoxide reductase complex subunit 1 (*VKORC1*) was first identified as part of the vitamin K epoxide reductase multiprotein complex (VKOR) in 2004 [1], [2]. The product of *VKORC1* is a rate-controlling enzyme in the vitamin cycle and is essential for the production of vitamin-K-dependent, γ-carboxylated proteins such as clotting factors II, VII, IX, X protein C, S, and Z. Thus, *VKORC1* has broad implications for clotting, a property well-appreciated: even before the gene was identified, VKOR has long been the target of warfarin, a commonly prescribed anticoagulant used to prevent stroke and other thromboembolic events. It is now known that rare mutations in *VKORC1* cause warfarin resistance, and common polymorphisms in *VKORC1* account a large proportion of the variability of warfarin dosing in most populations studied [3].

In addition to having broad effects on the coagulation cascade, the vitamin K cycle is also essential in the formation of the bone matrix. Vitamin K, which is synthesized by plants (K_1_) and bacteria in the gut (K_2_), is a required co-enzyme for the γ-carboxylation of three glutamic acid (Glu) residues in osteocalcin, converting them to gamma-carboxyglutamic acid (Gla). This post-translational Glu to Gla modification of osteocalcin, a bone and dentin protein produced by osteoblasts, is necessary for calcium binding. Evidence suggests that vitamin K_1_ deficiency is associated with decreased BMD [4], [5] and that high-dose vitamin K supplementation prevents fractures in at-risk patients [6]. Also, some inconsistent evidence suggests that long-term warfarin therapy, which by design inhibits the vitamin K cycle and prevents the Glu to Gla modification, is associated with low BMD in patients compared with patients not on warfarin-therapy [5], [7]. This latter observation in humans, however, is not supported by recent experiments in male rhesus macaques that demonstrate long-term warfarin therapy does not affect BMD while on a diet high in calcium and vitamin D [8].

Despite the discordant observations between humans and macaques, preliminary studies in humans suggest that *VKORC1* common variation is associated with mean undercarboyxlated osteocalcin [9] and dietary vitamin K intake [9], [10]. Based on role of *VKORC1* in the vitamin K cycle and based on the preliminary data presented in other studies, we hypothesized that common *VKORC1* genetic variation is associated with BMD in humans. To test this hypothesis, we genotyped six *VKORC1* SNPs (rs9923231, rs9934438, rs2359612, rs8050894, rs2884737, rs7294) in the Third National Health and Nutrition Examination Survey (NHANES III) and tested for associations with measures of BMD. Four of these tagSNPs (rs9923231, rs9934438, rs2359612, and rs8050894) are known to be in strong linkage disequilibrium with one another and are associated with warfarin dosing in populations of European-descent [3], [11], [12]. We also tested for associations between *VKORC1* SNPs and osteoporosis, an extreme phenotype of BMD. Unadjusted and adjusted results suggest that *VKORC1* SNPs are associated with these bone phenotypes in human, but their effect size is likely small compared with other genetic and non-genetic factors.

## Materials and Methods

### Study Population

Participants were consented by the Centers for Disease Control and Prevention (CDC) at the time of the survey and sample collection, and consent included the storage of data and biological specimens such as blood for future research [13]. The present study was approved by the CDC Ethics Review Board. Because the study investigators did not have access to personal identifiers, this study was considered non-human subjects research by the Vanderbilt University Internal Review Board.

NHANES III was conducted between 1988 and 1994 by the National Center for Health Statistics (NCHS) at the CDC. NHANES is a nationally representative cross-sectional survey designed to represent non-institutionalized Americans at the time of ascertainment [14], [15]. NHANES is also a complex, multi-stage survey that oversamples minorities (non-Hispanic blacks and Mexican-Americans), children, and the elderly. Sampling weights are calculated and provided for analysis to account for non-response bias and to adjust for the oversampling of specific groups so that all estimates are nationally representative. All participants were asked to complete a household interview and physical examination in the Mobile Examination Center (MEC). If the participant was not able to visit the MEC, a home examination was arranged. During Phase 2 of NHANES III (1991–1994), cell lines were established from blood samples of participants >12 years of age. The total number of NHANES III phase 2 participants was 16,530, and sample weights were recalculated using methods previously described [16] for participants with DNA samples to avoid non-response bias. NHANES III DNA samples became available to study investigators beginning in 2002 [13], [17]–[19].

BMD of the proximal femur was measured during the physical exam on non-pregnant female and male participants at least 20 years of age using dual energy x-ray absorptiometry (DXA) [20]. Bone mineral content and BMD are available for the femur neck region (gm/cm^2^), the trochanter region (gm/cm^2^), the intertrochanter region (gm/cm^2^), the Ward's triangle region (gm/cm^2^), and the total region (gm/cm^2^). Cotinine levels were determined in participants using STC Diagnostics cotinine enzyme immunoassay (EIA) kits (Bethlehem, PA). Serum vitamin D levels were determined in participants using the DiaSorin radioimmunoassay (RIA) kit (formerly the INCSTAR 25-OH-D assay; Stillwater, MN) [21].

### Genotyping

NHANES III DNA samples were distributed as aliquots of crude cell lysates to study investigators. NHANES III DNA concentrations vary and are estimated to range from 7.5–65 ng/µL with an average of approximately four micrograms in 100 ul. NHANES III DNA samples are distributed in 96-well plates along with four 96-well plates of CDC-supplied blinded duplicates and blank controls. NHANES III experimental DNA samples are randomly distributed across plates without regard to race/ethnicity, sex, or case/control status. NHANES III DNA samples represent several major racial/ethnic groups, including non-Hispanic whites (n = 2,631), non-Hispanic blacks (n = 2,018), Mexican-Americans (n = 2,073), and other racial/ethnic groups (n = 437).

TagSNPs were selected using LDselect [22] and the MultiPop-TagSelect algorithm [23] as previously described [3] for non-Hispanic whites and non-Hispanic blacks. A total of 16 tagSNPs were considered for genotyping. *VKORC1* rs17880887 could not be successfully converted into a genotyping assay and was omitted from further genotyping attempts. Five tagSNPs were targeted for genotyping because they represent the vast majority of common variation in European-descent populations [3]. These five tagSNPs also represent the haplotypes associated with warfarin dosing in both non-Hispanic whites and non-Hispanic blacks [12], [24]. A sixth SNP (rs9923231), which is redundant with rs9934438 in both non-Hispanic whites and non-Hispanic blacks, was targeted for genotyping given that there is evidence this is the functional SNP in the association with warfarin dosing [11], [25].

A total of six SNPs were genotyped in 7,159 DNA samples in NHANES III: rs9923231, rs9934438, rs8050894, rs2359612, rs2884737, and rs7294 (Table 1 and Table S1). All SNPs were genotyped using Applied Biosystem's TaqMan® SNP Genotyping Assays (Foster City, CA) except for rs2884737, which was genotyped using Sequenom's iPLEX® Gold coupled with MassARRAY MALDI-TOF MS detection (San Diego, CA). The SNP genotyping call rates ranged from 90% to 99%, with an average call rate of ∼95%. All SNPs were in Hardy Weinberg Equilibrium (HWE) at p>0.05, and all SNPs passed CDC quality control measures based on tests of HWE on the experimental DNA samples and 368 blinded duplicates on CDC-supplied control plates. All genotypes have been deposited into CDC's Genetic NHANES database and are available for secondary analysis.

**Table 1:** *VKORC1* SNP alleles, SNP location, and minor allele frequency by race/ethnicity.

		Genotype frequencies		
		(minor allele frequency)		
SNP	SNP Location	Non-Hispanic white	Non-Hispanic black	Mexican-American
(major allele/minor allele)		(n = 2,631)	(n = 2,108)	(n = 2,073)
rs9923231	5′ flanking	0.38/0.47/0.15	0.81/0.18/0.01	0.30/0.50/0.20
(G/A)		(0.38)	(0.10)	(0.45)
rs2884737	5′ flanking	0.56/0.37/0.07	0.91/0.08/0.01	0.74/0.24/0.02
(A/C)		(0.26)	(0.05)	(0.49)
rs9934438	Intronic	0.38/0.47/0.15	0.81/0.18/0.0.01	0.31/0.49/0.20
(G/A)		(0.38)	(0.10)	(0.45)
rs8050984	Intronic	0.34/0.50/0.17	0.49/0.42/0.09	0.25/0.51/0.23
(C/G)		(0.42)	(0.30)	(0.49)
rs2359612	Intronic	0.39/0.47/0.14	0.65/0.31/0.04	0.30/0.50/0.20
(G/A)		(0.38)	(0.20)	(0.45)
rs7294	3′ untranslated region	0.39/0.48/0.13	0.31/0.49/0.19	0.36/0.49/0.16
(C/T)		(0.37)	(0.19)	(0.40)

### Statistical Analysis

All analyses were conducted remotely in SAS v9.2 (SAS Institute, Cary, NC) and SUDAAN (Research Triangle Institute, Research Triangle Park, NC) using the Analytic Data Research by Email (ANDRE) portal of the CDC Research Data Center in Hyattsville, MD. All analyses presented here were performed weighted. Unweighted analyses were not substantially different compared with weighted analyses (data not shown).

Linear regressions stratified by sex and race/ethnicity were performed where BMD was the dependent variable. Models were adjusted for the following variables: age (in years; continuous), body mass index (kg/m^2^; continuous), current smoking status (defined by “do you smoke cigarettes now?” or cotinine levels >15 ng/ml; binary); family history of osteoporosis (“Doctor told mother she had osteoporosis”; binary), thyroid disease (“Doctor ever told you had thyroid disease”; binary), menopause (defined as a woman >60 years of age answering “no” to “have you had a period in the past 12 months” or as a woman with bilateral oophorectomy answering “yes” to “have you had one or both ovaries removed” and “both removed”; binary); hysterectomy (“have you had a hysterectomy”; binary), education (defined as less than high school, high school, and greater than high school from “highest grade or year completed”; categorical), use of hormone replacement therapy (defined as “ever/never” from three questions: “ever take estrogen by mouth,” “have you ever taken or used estrogen or female hormones in the form of vaginal cream,” and “have you ever used female hormones in the form of patches that are placed on the skin”; binary), and oral contraceptive use (“have you ever taken birth control pills for any reason?”; binary). Dietary variables such as calcium (mg; continuous) and alcohol consumption (gm; continuous) were defined from the 24-hour dietary recall.

Logistic regression was performed where osteoporosis was the dependent variable. Osteoporosis was defined as less than or equal to −2.5 standard deviations from the mean BMD total region. The mean BMD used to define cases and controls is based on participants 20–29 years of age in each sex and race/ethnicity group, which is based on the criteria outlined by WHO in 1994 (as described in [26]). We adjusted models using the same variables from the linear regression.

SNPs were included in both the linear and logistic regression models assuming an additive genetic model (genotypes coded as 0, 1, and 2). SNPs were first included in the model without adjustment and then included in the fully adjusted models.

## Results

The study population characteristics are given in Table 2. For each *VKORC1* SNP, unadjusted tests of association for BMD total region were performed assuming additive genetic model stratified by race/ethnicity and sex (Table 3). Among non-Hispanic black males, two SNPs were significantly associated with increased BMD (rs9923231, p = 0.015 and rs9934438, p = 0.004), and one SNP was significantly associated with decreased BMD (rs8050894, p = 0.014). One SNP, rs7294, was associated with decreased BMD among non-Hispanic white males (p = 0.011). No significant associations were identified in non-Hispanic white females, non-Hispanic black females, or Mexican American males or females.

**Table 2:** Study population characteristics for participants ≥20 years of age stratified by race/ethnicity and sex.

	Non-Hispanic whites		Non-Hispanic blacks		Mexican-Americans	
	*Females*	*Males*	*Females*	*Males*	*Females*	*Males*
	(n = 1,327)	(n = 884)	(n = 809)	(n = 619)	(n = 726)	(n = 795)
**Mean age (years)**	47.17	45.17	42.46	41.38	38.43	36.95
	(1.12)	(1.01)	(0.86)	(0.79)	(0.75)	(0.80)
**Mean BMI (kg/m^2^)**	26.26	27.04	29.42	26.77	28.54	27.12
	(0.32)	(0.17)	(0.28)	(0.26)	(0.19)	(0.19)
**Current smokers (%)**	26.33	36.73	33.76	44.67	16.88	32.78
**Family history of maternal osteoporosis (%)**	6.86	2.88	1.68	1.19	2.72	1.44
**Thyroid disease (%)**	9.66	2.67	3.35	1.04	3.78	0.96
**Menopause (%)**	24.11	n/a	8.62	n/a	9.58	n/a
**Hysterectomy (%)**	25.25	n/a	19.50	n/a	15.01	n/a
**Oral contraceptive use (%)**	61.84	n/a	61.93	n/a	58.76	n/a
**Hormone replacement therapy (%)**	21.27	n/a	12.32	n/a	8.61	n/a
**High school education (%)**	56.86	49.89	62.89	59.70	48.46	42.21
**Mean alcohol consumption (gm)**	5.83	14.69	5.03	18.04	3.33	19.25
	(0.68)	(1.94)	(0.28)	(1.69)	(0.78)	(1.48)
**Mean dietary calcium (mg)**	741.70	1074.43	546.71	786.92	754.58	972.51
	(19.21)	(36.02)	(15.29)	(24.17)	(21.33)	(16.95)
**Mean serum vitamin D (ng/mL)**	31.46	33.46	17.48	20.21	23.22	28.32
	(0.59)	(0.63)	(0.60)	(0.80)	(0.50)	(0.75)
**Mean total region BMD (gm/cm^2^)**	0.87	0.99	0.98	1.11	0.93	1.04
	(0.01)	(0.01)	(0.01)	(0.02)	(0.01)	(0.01)

Weighted means (standard errors of the mean) and proportions are provided for each variable. Sample sizes given are based on the counts available for total bone mineral density.

Abbreviations: Body mass index (BMI), bone mineral density (BMD).

**Table 3:** Unadjusted and weighted single SNP tests of associations, by race/ethnicity and sex, for bone mineral density total region (gm/cm^2^).

	Non-Hispanic whites				Non-Hispanic blacks				Mexican-Americans			
	*Females*		*Males*		*Females*		*Males*		*Females*		*Males*	
SNP	Beta	p-value	Beta	p-value	Beta	p-value	Beta	p-value	Beta	p-value	Beta	p-value
	(SE)		(SE)		(SE)		(SE)		(SE)		(SE)	
rs9923231	<0.01	0.837	0.01	0.370	0.01	0.473	0.01	***0.015***	<−0.01	0.604	<0.01	0.689
	(0.01)		(0.01)		(0.01)		(0.02)		(0.01)		(0.01)	
rs9934438	<0.01	0.860	0.01	0.440	0.01	0.550	0.05	***0.004***	<−0.01	0.733	0.01	0.487
	(0.01)		(0.01)		(0.01)		(0.02)		(0.01)		(0.01)	
rs8050894	<−0.01	0.969	−0.01	0.426	0.01	0.408	−0.04	***0.014***	0.01	0.506	−0.01	0.543
	(0.01)		(0.01)		(0.01)		(0.01)		(0.01)		(0.01)	
rs2359612	<0.01	0.799	0.01	0.530	0.01	0.700	0.02	0.317	<−0.01	0.783	<0.01	0.721
	(0.01)		(0.01)		(0.01)		(0.02)		(0.01)		(0.01)	
rs2884737	−0.01	0.270	<−0.01	0.665	0.03	0.082	−0.05	0.167	−0.01	0.606	−0.02	0.097
	(0.01)		(0.01)		(0.02)		(0.03)		(0.01)		(0.01)	
rs7294	0.01	0.145	−0.03	***0.011***	0.01	0.491	<−0.01	0.965	<−0.01	0.700	<−0.01	0.491
	(<0.01)		(0.01)		(0.01)		(0.01)		(0.01)		(0.01)	

Adjustment for age, body mass index, smoking status, maternal family history of osteoporosis, thyroid disease, menopause, hysterectomy, oral contraceptive use, hormone replacement therapy, education level, alcohol consumption, dietary calcium and vitamin K, and serum levels of vitamin D did not appreciably alter the associations observed in the unadjusted analyses (Table 4). That is, SNPs rs9923231 and rs9934438 were both associated with increased BMD (p = 0.039 and 0.024) and rs8050894 was associated with decreased BMD among non-Hispanic black males (p = 0.016). *VKORC1* SNP rs7294 was no longer associated among non-Hispanic white males. SNP rs2884737, which was not significant in unadjusted models, was significantly associated with decreased BMD among Mexican-American males (p = 0.004).

**Table 4:** Adjusted and weighted single SNP tests of associations, by race/ethnicity and sex, for bone mineral density total region (gm/cm^2^).

	Non-Hispanic whites				Non-Hispanic blacks				Mexican-Americans			
	*Females*		*Males*		*Females*		*Males*		*Females*		*Males*	
SNP	Beta	p-value	Beta	p-value	Beta	p-value	Beta	p-value	Beta	p-value	Beta	p-value
	(SE)		(SE)		(SE)		(SE)		(SE)		(SE)	
rs9923231	<0.01	0.400	<0.01	0.622	<−0.01	0.743	0.03	***0.039***	<−0.01	0.283	0.01	0.393
	(0.01)		(0.01)		(0.02)		(0.01)		(0.01)		(0.01)	
rs9934438	<0.01	0.321	<0.01	0.847	<−0.01	0.757	0.03	***0.024***	<−0.01	0.513	0.01	0.275
	(0.01)		(0.01)		(0.02)		(0.01)		(0.01)		(0.01)	
rs8050894	<−0.01	0.630	−0.01	0.497	0.01	0.393	−0.03	***0.016***	0.02	0.219	−0.01	0.403
	(<0.01)		(0.01)		(0.02)		(0.01)		(0.01)		(0.01)	
rs2359612	<0.01	0.460	<0.01	0.830	<−0.01	0.487	0.01	0.518	<−0.01	0.363	0.00	0.560
	(0.01)		(0.01)		(0.01)		(0.01)		(0.01)		(0.01)	
rs2884737	<0.01	0.864	<−0.01	0.703	0.01	0.643	−0.04	0.138	0.01	0.473	−0.03	***0.004***
	(0.01)		(0.01)		(0.03)		(0.02)		(0.01)		(0.01)	
rs7294	0.01	0.154	−0.02	0.066	<0.01	0.238	−0.00	0.736	<0.01	0.647	−0.01	0.192
	(<0.01)		(0.01)		(0.01)		(0.01)		0.01		(0.01)	

Single SNP test of association are adjusted by variables given in Table 2.

Given that *VKORC1* SNPs were associated with BMD total region, we tested whether *VKORC1* SNPs were associated with osteoporosis. In unadjusted tests of association, only rs7294 was associated with osteoporosis. This significant association (p = 0.001) was observed only among non-Hispanic white males (odds ratio  = 0.60; 95% confidence interval  = 0.45, 0.79; Table S2). After adjustment for age, body mass index, smoking status, maternal family history of osteoporosis, thyroid disease, education level, alcohol consumption, dietary calcium and vitamin K, and serum levels of vitamin D, the association between rs7294 and osteoporosis among non-Hispanic white males remained significant (p = 0.04; OR = 0.65; 95% CI  = 0.44, 0.98; Table S3). Adjusted models also revealed a significant association not observed in unadjusted analyses (Table S3). Specifically, rs8050894 was associated with osteoporosis in Mexican-American males (p = 0.03; OR  = 1.40; 95% CI  = 1.04, 1.87).

## Discussion

We genotyped six SNPs in the candidate gene *VKORC1* in 7,159 participants of NHANES III to determine if these common genetic variants contribute to the variability in BMD in the general population. Previous studies suggested that the vitamin K cycle is essential to the formation of the bone matrix. Furthermore, patients on long-term warfarin therapy, of which *VKORC1* is the target, have on average lower BMD compared with those not on long term warfarin [7]. Our results suggest that common variants in *VKORC1* are indeed associated with BMD and perhaps osteoporosis, but many of these results are limited to African Americans. Also, the *VKORC1* SNPs, while associated at p<0.05, contribute very little to variability of BMD (<1%) compared with other risk factors, making it unlikely that this locus is a major contributor to BMD as a main effect.

The weak contribution of *VKORC1* SNPs on BMD and osteoporosis is not surprising given that BMD and osteoporosis are complex traits likely influenced by both genetics and the environment. Twin and family studies suggest 40–80% of the variability observed in BMD in various study populations can be attributable to genetics [27]–[33]. Likewise, for osteoporosis, a family history of the condition is strongly associated with cases compared with controls [34], [35]. To date, the genetic component described in these twin and family studies seems to consist of many common genetic variants, each with very small effects. That is, candidate gene [36]–[39] and genome-wide association studies [28], [40]–[46] have identified >20 genes or genomic regions associated with hip and spine BMD and/or osteoporosis, each with effect sizes explaining <1 to 4% of the variability in BMD or with an odds ratio of <1.5 for osteoporosis.

This is the first report of an association between BMD and osteoporosis and these *VKORC1* SNPs in the literature. *VKORC1* genetic variation on chromosome 16 is not in linkage disequilibrium with genetic variation known to be associated with BMD (such as *ESR1* variants on chromosome 6 [47]) through GWAS and candidate gene studies. Thus, the associations reported here could represent false-positive findings or could represent associations that fall below the accepted threshold for significance in genome-wide association studies (p<5.0×10^−8^). It is interesting to note, however, that our associations in BMD are mostly limited to African American males. To date, few GWAS studies have been performed in populations of non-European descent for BMD or osteoporosis, and none have been reported for populations of African-descent. This latter situation has an impact on our ability to replicate the associations described here as GWA studies available in dbGaP [48], the public repository for genotypes and phenotypes, are not from populations of similar genetic ancestry (i.e., the Framingham Heart Study is of European-descent). For early replication studies, the preferred sequence of events is to first replicate and confirm associations in populations of similar genetic ancestry before performing characterization studies in other racial/ethnic populations [49].

Indeed, differences in genetic variation and linkage disequilibrium patterns may explain, in part, the population-specific associations described here. As already previously described [3], [50], the linkage disequilibrium pattern in *VKORC1* differs between European-descent and African-descent populations, with the latter having less pair-wise LD. Four of the six SNPs (rs9923231, rs9934438, rs8050894, and rs2359612) genotyped in NHANES III are in strong LD in the non-Hispanic white subpopulation, but only two (rs9923231 and rs9934438) are in LD in the non-Hispanic black subpopulation. Intronic rs8050894 is not in LD with other genotyped *VKORC1* SNPs in non-Hispanic blacks and was associated with decreased BMD in males. This SNP, along with the three in LD with it, was not associated with BMD in the non-Hispanic white population. It is possible this independent association observed in non-Hispanic black males is tagging a genetic variant not genotyped in this study that is more common in African-descent populations compared with European-descent populations. In contrast, the two SNPs in LD in non-Hispanic black males were associated with increased BMD, but, again, neither these SNPs nor the two SNPs in LD with them were associated with BMD in non-Hispanic whites. The lack of association observed in non-Hispanic whites is less straightforward given that experimental evidence suggests 5′ flanking rs9923231 affects *VKORC1* gene expression [25]. Nevertheless, it is still possible that the association with increased BMD in non-Hispanic blacks also represents an unknown genetic variant tagged by rs9923231 and rs9934438.

In relation to *VKORC1*'s association with warfarin dosing, it is interesting that the minor alleles of rs9923231 and rs9934438 are associated with increased BMD in non-Hispanic black males. The minor alleles of these two *VKORC1* SNPs are also associated with decreased warfarin dose compared with the major alleles [11]. And, several studies have shown that these *VKORC1* minor alleles are associated with decreased *VKORC1* expression in liver [12], [25]. NHANES III participants are drawn from the general population, so the relationship between warfarin dose, BMD, and *VKORC1* could not be directly assessed in this population.

Of note, also, is the sex-specific nature of the associations described here. It is already known that mean BMD differs by both sex and race/ethnicity [20], [51], and sex differences are also supported in mouse models [52]. Additionally, previous segregation, linkage, and association studies support sex-specific genetic effects for BMD [53], [54] and osteoporosis-related fractures [37]. It is unlikely that power explains the lack of associations observed among non-Hispanic black females given that the sample size for this subgroup (n = 809) is larger than the non-Hispanic black male subgroup (n = 619). Also, in adjusted analyses, we included the same demographic and dietary variables in all sex-specific models, with the only differences related to female-only variables (such as menopause, oral contraceptive use, and hormone replacement therapy). We cannot rule out the possibility that unknown variables (confounders) are responsible for the observed associations in males only; nevertheless, the sex-specific effects are intriguing and warrant further study.

This is a large, population-based study of a diverse sample from the United States. Despite the strength of sample size for BMD, this study has several limitations. First, the age range of the study is wide, as participants in NHANES III aged 12 years and greater are available for Genetic NHANES III, and those ≥20 years have BMD measurements available. Attempts to examine older adults with BMD are hampered by small sample sizes within any one subgroup, as evidenced by the small number of cases of osteoporosis. Second, our study is a candidate gene study and necessarily limited compared with genome-wide association studies. Third, we did not adjust for multiple comparisons using Bonferroni correction given this method is conservative when SNPs are linkage disequilibrium with one another [55]. Even if we chose to adjust using Bonferroni, it is not clear how to implement this correction given each subpopulation has a distinct pattern of linkage disequilibrium for this candidate gene [3]. Therefore, we present here unadjusted p-values. Finally, Genetics NHANES III does not have ancestry informative markers or GWAS data available to adjust for population stratification. We used self-reported race/ethnicity to stratify NHANES prior to analysis. While population stratification may still be a concern in this study, it is worth noting that previous studies have found self-reported race/ethnicity is highly concordant with genetic ancestry determined by genetic markers [56].

In conclusion, we describe several sex- and race/ethnic-specific associations between BMD and *VKORC1* SNPs in adults ascertained for a large, population-based cross-sectional survey of the United States. This is the first report of *VKORC1* SNPs associated with BMD; therefore, further studies are required to replicate and characterize the association to establish this candidate gene as a locus relevant to BMD and perhaps associated phenotypes such as osteoporosis.

## Supporting Information

Table S1 **Pair-wise linkage disequilibrium (r^2^) for NHANES III participants genotyped for six** ***VKORC1*** **SNPs, by populations.**
(DOCX)

Table S2 **Unadjusted and weighted single SNP tests of association, by race/ethnicity and sex, for osteoporosis.** Odds ratios (95% confidence intervals) are presented.
(DOCX)

Table S3 **Adjusted and weighted single SNP tests of association, by race/ethnicity and sex, for osteoporosis.** Single SNP tests of association were adjusted for variables in Table 2. Odds ratios (95% confidence intervals) are presented.
(DOCX)
