# Supplementary material for: VKORC1 Common Variation and Bone Mineral Density in the Third National Health and Nutrition Examination Survey
Source: PLoS One. 2010 Dec 13;5(12):e15088. doi: 10.1371/journal.pone.0015088 (PMC3001474; doi:10.1371/journal.pone.0015088)
Supplement: Table S1 — Pair-wise linkage disequilibrium (r2) for NHANES III participants genotyped for six VKORC1 SNPs, by populations. (DOCX) [file pone.0015088.s001.docx]

**Table S1. Pair-wise linkage disequilibrium (r^2^) for NHANES III participants genotyped for six *VKORC1* SNPs, by populations**

| ***non-Hispanic whites*** | | | | | | |
| --- | --- | --- | --- | --- | --- | --- |
|  | **rs7294** | **rs2359612** | **rs9934438** | **rs2884737** | **rs9923231** | **rs8050984** |
| **rs7294** | 1.000 | 0.347 | 0.347 | 0.193 | 0.349 | 0.350 |
| **rs2359612** | 0.966 | 1.000 | 0.987 | 0.551 | 0.992 | 0.925 |
| **rs9934438** | 0.963 | 0.994 | 1.000 | 0.551 | 0.992 | 0.929 |
| **rs2884737** | 0.967 | 0.999 | 0.999 | 1.000 | 0.554 | 0.511 |
| **rs9923231** | 0.966 | 0.999 | 0.998 | 0.999 | 1.000 | 0.935 |
| **rs8050984** | 0.928 | 0.995 | 0.997 | 1.000 | 1.000 | 1.000 |
| ***non-Hispanic blacks*** | | | | | | |
|  | **rs7294** | **rs2359612** | **rs9934438** | **rs2884737** | **rs9923231** | **rs8050984** |
| **rs7294** | 1.000 | 0.196 | 0.090 | 0.037 | 0.091 | 0.279 |
| **rs2359612** | 0.991 | 1.000 | 0.452 | 0.186 | 0.450 | 0.074 |
| **rs9934438** | 0.989 | 0.990 | 1.000 | 0.401 | 0.981 | 0.281 |
| **rs2884737** | 1.000 | 1.000 | 0.987 | 1.000 | 0.408 | 0.113 |
| **rs9923231** | 1.000 | 0.997 | 0.995 | 1.000 | 1.000 | 0.280 |
| **rs8050984** | 0.917 | 0.353 | 1.000 | 1.000 | 0.996 | 1.000 |
| ***Mexican-Americans*** | | | | | | |
|  | **rs7294** | **rs2359612** | **rs9934438** | **rs2884737** | **rs9923231** | **rs8050984** |
| **rs7294** | 1.000 | 0.527 | 0.516 | 0.106 | 0.520 | 0.527 |
| **rs2359612** | 0.963 | 1.000 | 0.980 | 0.204 | 0.979 | 0.908 |
| **rs9934438** | 0.961 | 0.999 | 1.000 | 0.205 | 0.995 | 0.925 |
| **rs2884737** | 0.975 | 1.000 | 0.996 | 1.000 | 0.206 | 0.190 |
| **rs9923231** | 0.961 | 0.998 | 0.999 | 1.000 | 1.000 | 0.931 |
| **rs8050984** | 0.931 | 0.982 | 0.999 | 1.000 | 1.000 | 1.000 |
