# Supplementary material for: VKORC1 Common Variation and Bone Mineral Density in the Third National Health and Nutrition Examination Survey
Source: PLoS One. 2010 Dec 13;5(12):e15088. doi: 10.1371/journal.pone.0015088 (PMC3001474; doi:10.1371/journal.pone.0015088)
Supplement: Table S2 — Unadjusted and weighted single SNP tests of association, by race/ethnicity and sex, for osteoporosis. Odds ratios (95% confidence intervals) are presented. (DOCX) [file pone.0015088.s002.docx]

**Table S2. Unadjusted and weighted single SNP tests of association, by race/ethnicity and sex, for osteoporosis.** Odds ratios (95% confidence intervals) are presented.

|  | **Non-Hispanic whites** | | **Non-Hispanic blacks** | | **Mexican-Americans** | |
| --- | --- | --- | --- | --- | --- | --- |
|  | *Females* | *Males* | *Females* | *Males* | *Females* | *Males* |
|  | **(246 cases, 1,182 controls)** | **(75 cases, 861 controls)** | **(159 cases, 795 controls)** | **(56 cases, 614 controls)** | **(154 cases, 692 controls)** | **(48 cases, 786 controls)** |
| **rs9923231** | 1.04 | 1.16 | 0.98 | 1.39 | 1.25 | 0.94 |
|  | (0.79, 1.37) | (0.70, 1.94) | (0.64, 1.50) | (0.61, 3.20) | (0.96, 1.65) | (0.48, 1.84) |
| **rs9934438** | 1.06 | 1.18 | 1.00 | 1.39 | 1.24 | 0.97 |
|  | (0.79, 1.42) | (0.70, 1.97) | (0.65, 1.55) | (0.60, 3.20) | (0.94, 1.63) | (0.49, 1.91) |
| **rs8050894** | 0.96 | 0.85 | 0.83 | 0.61 | 0.78 | 1.34 |
|  | (0.76, 1.21) | (0.49, 1.49) | (0.59, 1.17) | (0.36, 1.04) | (0.57, 1.07) | (0.86, 2.10) |
| **rs2359612** | 1.04 | 1.12 | 1.13 | 1.18 | 1.22 | 0.90 |
|  | (0.79, 1.37) | (0.68, 1.83) | (0.82, 1.54) | (0.67, 2.08) | (0.92, 1.62) | (0.44, 1.85) |
| **rs2884737** | 1.04 | 0.78 | 1.18 | 0.36 | 1.13 | 1.62 |
|  | (0.78, 1.40) | (0.52, 1.16) | (0.64, 2.16) | (0.08, 1.66) | (0.68, 1.86) | (0.77, 3.40) |
| **rs7294** | 1.01 | ***0.60*** | 0.75 | 0.86 | 0.85 | 1.59 |
|  | (0.76, 1.34) | ***(0.45, 0.79)*** | (0.56, 1.01) | (0.52, 1.45) | (0.59, 1.23) | (0.90, 2.81) |
