# Supplementary material for: VKORC1 Common Variation and Bone Mineral Density in the Third National Health and Nutrition Examination Survey
Source: PLoS One. 2010 Dec 13;5(12):e15088. doi: 10.1371/journal.pone.0015088 (PMC3001474; doi:10.1371/journal.pone.0015088)
Supplement: Table S3 — Adjusted and weighted single SNP tests of association, by race/ethnicity and sex, for osteoporosis. Single SNP tests of association were adjusted for variables in Table 2. Odds ratios (95% confidence intervals) are presented. (DOCX) [file pone.0015088.s003.docx]

**Table S3. Adjusted and weighted single SNP tests of association, by race/ethnicity and sex, for osteoporosis.** Single SNP tests of association were adjusted for variables in Table 2. Odds ratios (95% confidence intervals) are presented.

|  | **Non-Hispanic whites** | | **Non-Hispanic blacks** | | **Mexican-Americans** | |
| --- | --- | --- | --- | --- | --- | --- |
|  | *Females* | *Males* | *Females* | *Males* | *Females* | *Males* |
|  | **(246 cases, 1,182 controls)** | **(75 cases, 861 controls)** | **(159 cases, 795 controls)** | **(56 cases, 614 controls)** | **(154 cases, 692 controls)** | **(48 cases, 786 controls)** |
| **rs9923231** | 1.13 | 0.96 | 1.44 | 1.30 | 1.07 | 0.94 |
|  | (0.78, 1.64) | (0.64, 1.43) | (0.67, 3.07) | (0.52, 3.26) | (0.45, 2.53) | (0.51, 1.75) |
| **rs9934438** | 1.16 | 0.95 | 1.46 | 1.30 | 1.05 | 1.00 |
|  | (0.78, 1.71) | (0.64, 1.41) | (0.68, 3.16) | (0.53, 3.20) | (0.44, 2.52) | (0.53, 1.86) |
| **rs8050894** | 0.88 | 1.05 | 0.57 | 0.72 | 0.89 | ***1.40*** |
|  | (0.65, 1.18) | (0.69, 1.59) | (0.30, 1.09) | (0.38, 1.33) | (0.34, 2.36) | ***(1.04, 1.87)*** |
| **rs2359612** | 1.15 | 0.93 | 1.17 | 1.21 | 1.00 | 0.91 |
|  | (0.79, 1.67) | (0.64, 1.34) | (0.65, 2.10) | (0.60, 2.47) | (0.42, 2.39) | (0.48, 1.73) |
| **rs2884737** | 0.96 | 0.91 | 1.13 | 0.49 | 0.93 | 1.80 |
|  | (0.63, 1.47) | (0.67, 1.24) | (0.31, 4.15) | (0.12, 1.90) | (0.51, 1.69) | (0.78, 4.16) |
| **rs7294** | 0.99 | ***0.65*** | 0.81 | 0.92 | 1.43 | 1.64 |
|  | (0.68, 1.42) | ***(0.44, 0.98)*** | (0.49, 1.35) | (0.58, 1.45) | (0.75, 2.70) | (0.90, 3.00) |
